# Analysis of the Osteogenic Effects of Biomaterials Using Numerical Simulation

**DOI:** 10.1155/2017/6981586

**Published:** 2017-01-02

**Authors:** Lan Wang, Jie Zhang, Wen Zhang, Hui-Lin Yang, Zong-Ping Luo

**Affiliations:** ^1^Orthopaedic Institute, Soochow University, Suzhou, China; ^2^Department of Fundamental Courses, Wuxi Institute of Technology, Wuxi, China; ^3^Department of Orthopaedics, The First Affiliated Hospital, Soochow University, Suzhou, China

## Abstract

We describe the development of an optimization algorithm for determining the effects of different properties of implanted biomaterials on bone growth, based on the finite element method and bone self-optimization theory. The rate of osteogenesis and the bone density distribution of the implanted biomaterials were quantitatively analyzed. Using the proposed algorithm, a femur with implanted biodegradable biomaterials was simulated, and the osteogenic effects of different materials were measured. Simulation experiments mainly considered variations in the elastic modulus (20–3000 MPa) and degradation period (10, 20, and 30 days) for the implanted biodegradable biomaterials. Based on our algorithm, the osteogenic effects of the materials were optimal when the elastic modulus was 1000 MPa and the degradation period was 20 days. The simulation results for the metaphyseal bone of the left femur were compared with micro-CT images from rats with defective femurs, which demonstrated the effectiveness of the algorithm. The proposed method was effective for optimization of the bone structure and is expected to have applications in matching appropriate bones and biomaterials. These results provide important insights into the development of implanted biomaterials for both clinical medicine and materials science.

## 1. Introduction

Biological systems have mechanisms to automatically adapt to environmental changes in order to maintain their required functions under varying conditions and stresses [1]. Many studies have investigated functional biological adaptations to environmental changes in the muscles, lungs [2, 3], arteries [4], bones [5, 6], and even plants [7]. For example, bones gradually adapt into an optimal structure to support our organs and human anatomical features. As the external loading changes, the bone structure will adopt an optimal shape and structure to adapt to the new loading; this process is called bone functional adaptation. The mechanisms mediating bone adaptation have been studied extensively. The “self-optimizing” properties of bone were described as early as 1896 [8], and a theory, known as Wolff's law, on the naturally optimum structure of bone was proposed.

Some of the earliest theoretical frameworks for bone adaptation included proposals for the elastic formulation of apparent bone density by an exponential penalization factor [9, 10]. Bendsøe [11] obtained similar results using topology optimization by the penalized material model. Recently, with the rapid development of computer technology, the quantitative study of bone functional adaptation has become possible [12]. Rossi and Wendling-Mansuy [13], Weinans et al. [14], and Beaupré et al. [15] combined mathematical descriptions with the finite element method (FEM) to quantitatively predict the self-optimizing features of bones, and the computed outcomes were shown to have many similarities with actual bone morphologies. Boyle and Kim [16] applied topology optimization to simulate three-dimensional human proximal femur remodeling and further proved the effectiveness of Wolff's hypothesis.

However, bones are not necessarily always healthy and may have defects or fractures. The rapid development of biomedicine has provided access to new methods such as implantation to support defective bones, stimulate cell growth, and release ions to help generate better bone structure. Moreover, the optimal features of bone materials, including compatibility, reactivity, and degradability, have been studied extensively for use in the clinical setting [17, 18].

For new bone, the initial mechanical strength is very important. If it is too strong, the concentration of stress on the new bone may be disadvantageous toward osteogenesis [19, 20]. If it is too weak, the bone cannot provide adequate support [21]. When choosing an optimal bone material, its initial biomechanical strength and its rate of degradation must be considered simultaneously [20]. If the degradation rate is too fast, voids in the bone may appear and lead to a weakened structure and the failure of osteogenesis [22]. If the rate is too slow, osteogenesis will be hindered [23]. Therefore, the appropriate bone material for implantation must be chosen to simultaneously satisfy both the degradation rate and new bone formation capacity, leading to the improved synchronization of both events. The implantation of bone material will cause changes in the stress applied to the bones, which will in turn cause changes in the internal structure optimization. The original optimization algorithm for bone cannot be used directly in defective bones, and the challenge thus becomes the matching of the bone material and osteogenesis. Thus, researchers in the fields of clinical research and related material sciences have begun to focus on the problem of matching the bone implantation material with the bone tissue [24].

The two existing research approaches for achieving this goal are experimental biomechanics and FEM analysis. Although constructing an experimental animal model has advantages such as intuitiveness and subjectivity [25], the need for long testing periods and susceptibility to numerous extraneous factors can be disadvantageous. This may limit the ability of researchers to observe any quantitative relationships between the implantation material and the bones. The current constitutive model plays an important role in understanding the effects of the implantation material on bone growth. Computational digital image processing technology and computer-aided analysis methods have simplified the creation of computer models [16, 21, 26]. We have preliminary achieved simulation of osteoblast with material and bone density distribution which considered the elastic modulus of material [27]. However, the degradation period has not been considered which was very important parameter for the implanted biodegradable biomaterials and has no animal experiments to provide the corresponding support.

In this study, we created a theoretical FEM model of bone remodeling with implanted biomaterials. Our bone growth optimization process takes into consideration Young's modulus and the degradation rate of the bone material, achieving a simulation of osteoblasts with material and bone density distributions. The simulation results for the metaphyseal bone of the rat left femur and micro-CT images from rats with experimental femur defects are compared. The results validate the effectiveness of the method in modeling bone structure and overall shape optimization. In addition, this method provides theoretical guidance to the matching problem between bones and implant biomaterials.

## 2. Materials and Methods

In this study, a combination of the bone self-adaptive optimization theory and material degradation rules was used to simulate the proximal femur with defects, as detailed below.

### 2.1. Finite Element Model

**Figure 1:**
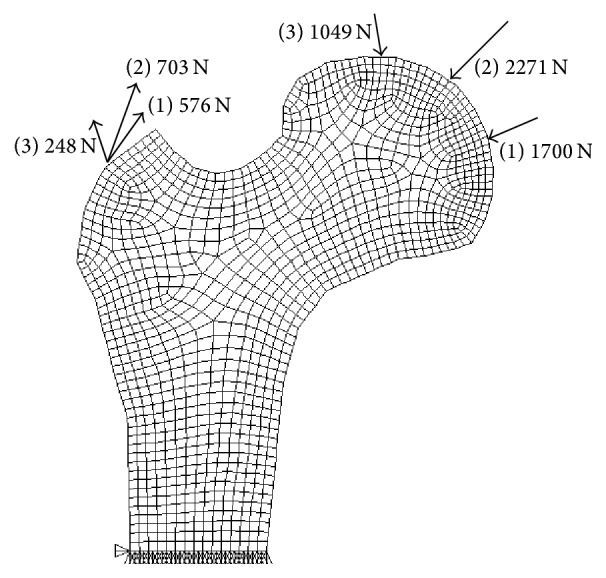
Finite element mesh for the proximal femur model.

A two-dimensional finite element model of the proximal femur was used. The model was obtained from the preprocessing of the femur of a normal adult male using CT, Photoshop 5.0, and ANSYS 10.0 software. With the ANSYS meshing tools, the model was divided into 3,689 nodes and 1,168 elements whose mesh element size was defined as 0.25 cm^2^ as shown in Figure 1. The implant material was linear, elastic, and isotropic. The material properties of the model included the elastic modulus *E* (2000 MPa), Poisson's ratio *μ* (0.3), and apparent density *ρ* (1.0 × 10^3^ kg·m^−3^) [13, 16]. The joint forces acting on the femoral head (different stress points) and the rotor muscle tendon tension were chosen based on the literature [28]. The loads are provided in Table 1. The defect was assumed to be 1 cm × 1 cm and involved 36 elements, including the cortical and cancellous bone. This size has been used in many prior animal experiments [25, 29, 30].

The ranges of implant material properties used in this study were previously defined [31, 32]. The elasticity modulus values were 30, 500, 1000, 2000, and 3000 MPa. For the material degradation time, the iteration step length was 1 day for the simulation. Three time periods (10, 20, and 30 days) were chosen.

### 2.2. Simulation-Based Analysis

The optimization of the objective function is presented as follows [27]:

(1) $$\begin{array}{c} F\left(\;\rho \left(\;t\;\right)\;\right)=\overset{n}{\underset{j=1}{\sum }}\left(\;\frac{1}{2}{\sigma_{j}\left(t\right)}^{T}S_{j}\left(\;t\;\right)\sigma_{j}\left(\;t\;\right)\;\right), \end{array}$$

where *F* is the strain energy at time *t*, *σ*_*j*_(*t*) is the stress vector component of element *j* at time *t*, *ρ*(*t*) = {*ρ*_*j*_(*t*)∣*j* = 1 ⋯ *n*}, *ρ*_*j*_(*t*) is the apparent density of element *j* at time *t*, *n* is the number of elements, and *S*_*j*_(*t*) is the global stiffness matrix, defined as follows:

(2) $$\begin{array}{c} S_{j}\left(\;t\;\right)=\left[\begin{array}{ccc} \frac{1}{E_{j}\left(t\right)} & -\frac{\mu_{j}\left(t\right)}{E_{j}\left(t\right)} & 0\\ -\frac{\mu_{j}\left(t\right)}{E_{j}\left(t\right)} & \frac{1}{E_{j}\left(t\right)} & 0\\ 0 & 0 & \frac{2\left(1+\mu_{j}\left(t\right)\right)}{E_{j}\left(t\right)} \end{array}\right], \end{array}$$

where *E*_*j*_(*t*) and *μ*_*j*_(*t*) represent the elastic modulus and Poisson's ratio, respectively.

The constraint equations are

(3) g1ρt=∑j=1nρjtvjt,

(4) 0<ρjt<1.8 g·cm−3,

where *g*_1_ was total mass constraint function and *v*_*j*_(*t*) is volume of element *j* at time *t*. Equation (3) indicates that the bone quality remains unchanged and (4) defines 1.8 g·cm^−3^ as the maximum density of the cortical bone during the optimization process. Through solving the optimization model, the density value of the next time was predicted to be $\hat{\rho }(t+1)=\left\{{\hat{\rho }}_{j}\left(t+1\right)|j=1\cdots n\right\}$.

Degradation of the material occurs slowly at earlier time points and more rapidly as time passes [30, 31]. In this study, an ideal material degradation function was established as follows: *E*1(*t* + 1) = (1 − *t*/*T*)^0.5^*∗E*1_0_, where *E*1_0_ is the initial modulus of the implant material (30, 500, 1000, 2000, and 3000 MPa) [31, 32], *E*1(*t* + 1) is the elastic modulus of the implant material at time *t* + 1, and *T* is degradation cycle. The relationship between the elastic modulus and apparent density is as follows: *E* = 2315*ρ*^3^ [21, 24].

The apparent densities of the defect elements and other part elements, respectively, follow the rules below:

(5) Δρit+1=c·ρ^it+1−ρit,i=nk  k is the implant material element,Δρkt+1=c·ρ^kt+1−ρkt−rt+1−rtk is the implant material element and k=1,…,36,

where *c* is the recycling control rate obtained from experience, defined as 0.02. *r*(*t*) was the degradation function of implant material and a second-order function was used in this work.

The next apparent density was calculated as follows:

(6) $$\begin{array}{c} \rho_{j}\left(\;t+1\;\right)=\rho_{j}\left(\;t\;\right)+\Delta \rho_{j}\left(\;t+1\;\right). \end{array}$$

The osteogenesis was the mean of the 36 implanted element which was obtained as *E*_*j*_ = 2315*ρ*_*j*_^3^ − *E*1(*j* + 1).

### 2.3. Animal Experiments and Finite Element Model

Seven 10-week-old female Sprague-Dawley rats with body weights of 204 ± 4 g were used for the micro-CT imaging study. The experimental protocol was approved by the Institutional Animal Committee. A defect (3 mm in diameter and 3 mm in depth) was drilled on the lateral side of the left femur. The biomaterial CSC (calcium sulfate cement), which has a degradation rate of 4–8 weeks in vivo for the rat model [33–36], was studied. The extents of biomaterial degradation and osteogenesis were scanned in vivo at three time points (7, 17, and 27 days) with a micro-CT scanner (SkyScan 1176, Bruker-microCT, Kontich, Belgium). The CSC, instrument parameters, and scanning methods were the same as the control animal experiment described in Zhang et al. [25], which restricted the rats in 47 × 35 × 20 cm^3^ cages.

Micro-CT images were reconstructed and the biggest horizontal truncation area of the refilled defect was calculated from the refilled defect volume by after-tracing the CSC edge visualized by the new bone formation, using the system software and a threshold of 110 in gray scale.

The simulation was designed on the basis of the animal experiments. The model, obtained using CT, Photoshop 5.0, and ANSYS 10.0 software, was based experimentally on the metaphyseal bone of the proximal left femur in rats. The implant material properties used were as follows: elastic moduli of 7000 and 2000 MPa and Poisson's ratio of 0.3 for two parts of the cortical bone; an elastic modulus of 900 MPa and Poisson's ratio of 0.3 for the cancellous bone; and an elastic modulus of 800 MPa and Poisson's ratio of 0.3 for the implant material. The joint force was assumed as 0.65 N, which is about one-third of an average rat body weight (204 g) [25]. For the material degradation time in the simulation, an iteration step length was 1 day.

## 3. Results

### 3.1. Initial State

**Figure 2:**
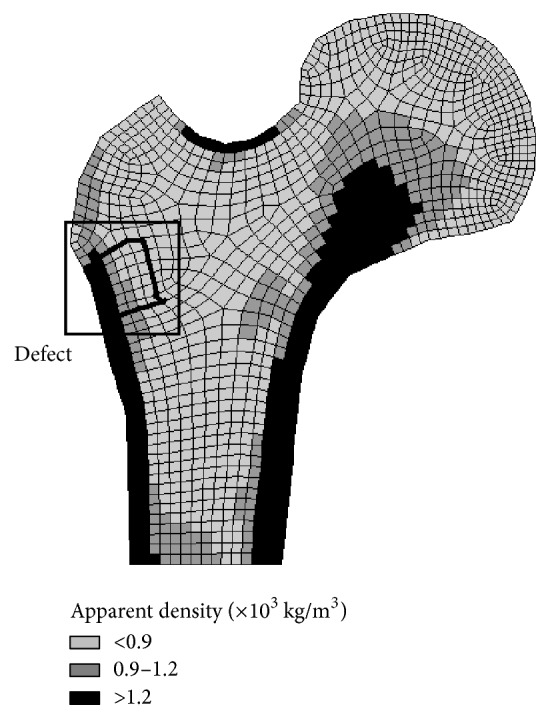
Density distribution for the proximal femur and defective area.

The initial proximal femur bone density distribution was obtained, as shown in Figure 2, based on the bone self-optimization theory [30, 31]. The darker gray region in the illustration represents a larger apparent density; the black area is the cortical bone, and the gray area is the cancellous bone. There are three types of apparent densities in the indicated square area black area that were chosen as defects. Thus, this model could be used to analyze the bone with the implant material.

**Figure 3:**
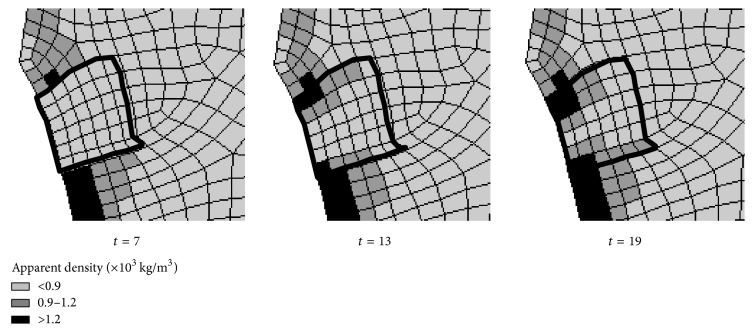
Apparent osteogenic density changes with *E* = 1000 MPa and *t* = 20 days.

The iterations were terminated after the density no longer exhibited significant change (value < 0.001) [30]. The step length was 1 day, which was set according to the material degradation and bone formation rates. An example of the apparent density change is given by Figure 3. Only the defective areas are shown because the apparent density was convergent and the rest of the structure exhibited nearly zero change. At day 7 after loading, the proportion of new bone formation is approximately zero. At days 17 and 19, the new bone formation proportions are 33.3% and 52.7%, respectively. As the biomaterial degrades, new bone is grown.

### 3.2. Effects of Material Properties and Degradation Frequency on Osteogenic Simulation

The impact of the average elastic moduli for different implant materials on osteogenesis is shown in [27]. The osteogenesis rate approaches zero when *E* = 0.002 MPa and the osteogenic effects are reduced compared with those of the stiffer material when *E* = 30 MPa. During the same degradation period of 20 days, the effects of the material with an elastic modulus of about 1000 MPa are improved compared with those of materials having either higher or lower elastic modulus values. Finally, at the highest simulated elastic modulus of 3000 MPa, the rate of osteogenesis is reduced because the material degrades more slowly and there is no space for new bone growth.

**Figure 4:**
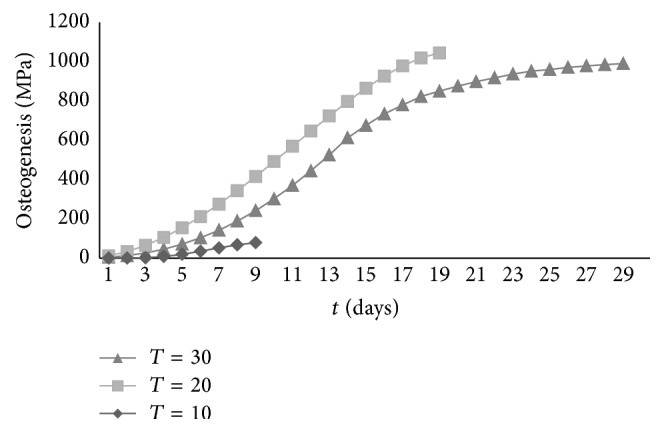
Contrast simulation results for osteogenesis using implant materials with different degradation periods.

Because optimal osteogenesis occurred at an elastic modulus of 1000 MPa, we used a material with this elastic modulus value to assess the effects of three degradation periods. The osteogenesis simulation results using implantation materials with different degradation periods at an elastic modulus of 1000 MPa are shown in Figure 4. Interestingly, we observe optimal osteogenesis when the degradation period is 20 days. When the degradation period is 10 days, degradation is too rapid, and the biomaterial cannot achieve maximum osteogenic effects. On the other hand, when the degradation period is 30 days, degradation is too slow, and there is no space for new bone to form.

### 3.3. Comparison with Animal Experiments

**Figure 5:**
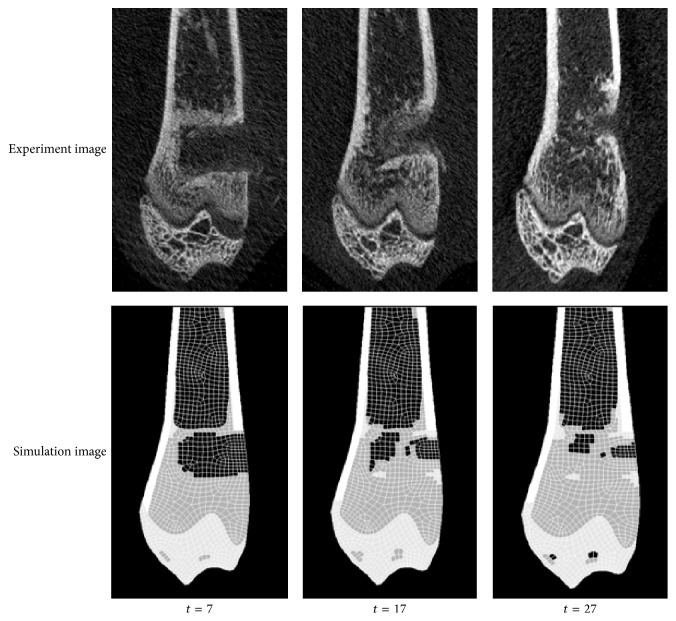
Micro-CT images from the animal experiments and simulation results showing the CSC degradation and new bone formation at *t* = 7, 17, and 27days.

The micro-CT images of the animal experiments and the simulation images showing the CSC degradation and new bone formation at *t* = 7, 17, and 27 days are presented in Figure 5. At day 7 after loading, the defect areas are 7.73 ± 0.41 and 8.21 mm^2^ for the control and the simulation, respectively. At day 17, the refilled defect areas are reduced in both the control (6.31 ± 0.43 mm^2^) and the simulation (6.70 mm^2^) and yet further reduced on day 27 (3.99 ± 0.35 and 4.01 mm^2^, respectively). The simulation results are well fitted to the experimental data.

## 4. Conclusions

Based on the theories and methods of Beaupré et al. [15, 24], we introduced a material degradation function which allowed us to develop methods that would numerically simulate and optimize bone growth after biomaterial implantation. These methods accurately described the bone density distribution after bone material implantation over time, thereby permitting us to propose theories and simulation techniques for bones in abnormal states. In this study, we verified that the implanted material may help defective bones to adapt to external loading and then quantitatively demonstrated that the bone adaptation to external loading had major effects on bone growth and followed Wolff's law.

The computer simulation provided a quantitative analysis method for resolving two basic but complementary problems between an implant material and bone growth: (1) quantifying the effects of the implant material and (2) finding a suitable biodegradable material. Compared to using animal experiments, we could reduce a 30-day study period to several hours via the computer simulation. Although degradation functions cannot precisely model how implant materials will behave, the simulation process uses discretized data with points chosen from journal references.

For simulation purposes, the degradation rate of the implant material was assumed to be constant; this may be achieved experimentally by using porous materials. However, due to the use of composite materials and the irregular shape of an implant, degradation will not be uniform across the bulk of the implant material. As a result, the next step will be to perform a prospective study based on the stimulation results, considering properties such as the dimensions, proportions, and structure of the material. Assuming that the material is isotropic simplifies the quantitative analyses of material degradation and bone growth; however, the anisotropic properties of the material will need to be considered later. Based on these studies, a more accurate and reasonable degradation analysis can be established by introducing more parameters into the degradation distribution function which will better fit the experimental results.

Application of the bone remodeling theory aided by computationally iterated simulation allows for the calculation of bone density, osteogenesis, and the development of defective bones. This may be helpful for planning during surgery and for the prediction of postoperative bone growth. Although these bone remodeling simulations are still rudimentary, bone remodeling theories are expected to become more refined as our understanding of 3D modeling and complex loading is improved. The applications of these data will be broad, particularly after improvement of the numerical simulation technology.

## Figures and Tables

**Table 1:** Load results for the proximal femur model.

	Joint reaction force/N	Angle/°	Rotor rally/N	Angle/°
1	1700	25	576	35
2	2271	66	703	62
3	1049	15	248	8

Note: the angle is the angle between the direction of force and the horizontal direction.

## References

[1] Florio C. S. (2015). Development of a widely applicable gradientless shape optimization based bone adaptation model for comparative parametric studies. *Structural and Multidisciplinary Optimization*.

[2] Campbell N. A. (1993). *Biology*.

[3] Cowan M. J., Crystal R. G. (1975). Lung growth after unilateral pneumonectomy: quantitation of collagen synthesis and content. *American Review of Respiratory Disease*.

[4] Fung Y. C. (1990). *Biomechanics: Motion, Flow, Stress, and Growth*.

[5] Roesler H. (1987). The history of some fundamental concepts in bone biomechanics. *Journal of Biomechanics*.

[6] Cowin S. C. (2008). *Bone Mechanics Handbook*.

[7] Archer R. R., Wilson B. F. (1970). Mechanics of the compression wood response. *Plant Physiology*.

[8] Wolff J. (1986). *The Law of Bone Remodeling [Translated from the 1892 Original, Das Gesetz der Transformation der Knochen, by P. Maquet and R. Furlong]*.

[9] Carte D. R., Hayes W. C. (1977). The behavior of bone as a two-phase porous structure. *Journal of Bone and Joint Surgery A*.

[10] Carter D. R. (1984). Mechanical loading histories and cortical bone remodeling. *Calcified Tissue International*.

[11] Bendsøe M. P. (1989). Optimal shape design as a material distribution problem. *Structural Optimization*.

[12] Hayes W. C., Snyder B., Cowin S. C. (1981). Toward a quantitative formulation of Wolff's law in trabecular bone. *Mechanical Properties of Bone*.

[13] Rossi J.-M., Wendling-Mansuy S. (2007). A topology optimization based model of bone adaptation. *Computer Methods in Biomechanics and Biomedical Engineering*.

[14] Weinans H., Huiskes R., Grootenboer H. J. (1992). The behavior of adaptive bone-remodeling simulation models. *Journal of Biomechanics*.

[15] Beaupré G. S., Orr T. E., Carter D. R. (1990). An approach for time‐dependent bone modeling and remodeling—theoretical development. *Journal of Orthopaedic Research*.

[16] Boyle C., Kim I. Y. (2011). Three-dimensional micro-level computational study of Wolff's law via trabecular bone remodeling in the human proximal femur using design space topology optimization. *Journal of Biomechanics*.

[17] Zins J. E., Moreira-Gonzalez A., Parikh A., Arslan E., Bauer T., Siemionow M. (2008). Biomechanical and histologic evaluation of the Norian craniofacial repair system and Norian Craniofacial Repair System Fast Set Putty in the long-term reconstruction of full-thickness skull defects in a sheep model. *Plastic and Reconstructive Surgery*.

[18] Zhao J., Shen G., Liu C. (2012). Enhanced healing of rat calvarial defects with sulfated chitosan-coated calcium-deficient hydroxyapatite/bone morphogenetic protein 2 scaffolds. *Tissue Engineering Part A*.

[19] Sikavitsas V. I., Temenoff J. S., Mikos A. G. (2001). Biomaterials and bone mechanotransduction. *Biomaterials*.

[20] Lu X., Leng Y. (2005). Theoretical analysis of calcium phosphate precipitation in simulated body fluid. *Biomaterials*.

[21] Stadelmann V. A., Conway C. M., Boyd S. K. (2013). In vivo monitoring of bone-implant bond strength by microCT and finite element modelling. *Computer Methods in Biomechanics and Biomedical Engineering*.

[22] Cao C.-F., Zhou J.-J., Pang J.-H., Chen X.-Q. (2011). A five-year clinical and radiographic follow-up of bipolar hip arthroplasty with insertion of a porous-coated anatomic femoral component without cement. *Orthopaedic Surgery*.

[23] Kihara H., Shiota M., Yamashita Y., Kasugai S. (2006). Biodegradation process of *α*-TCP particles and new bone formation in a rabbit cranial defect model. *Journal of Biomedical Materials Research—Part B Applied Biomaterials*.

[24] Beaupré G. S., Orr T. E., Carter D. R. (1990). An approach for time‐dependent bone modeling and remodeling—application: a preliminary remodeling simulation. *Journal of Orthopaedic Research*.

[25] Zhang J., Wang L., Zhang W., Zhang M., Luo Z.-P. (2015). Synchronization of calcium sulphate cement degradation and new bone formation is improved by external mechanical regulation. *Journal of Orthopaedic Research*.

[26] Annicchiarico W., Martinez G., Cerrolaza M. (2007). Boundary elements and *β*-spline surface modeling for medical applications. *Applied Mathematical Modelling*.

[27] Lan W., Wen Z., Hui-Lin Y., Zong-Ping L. Osteogenesis effect of biomaterials analyzed using topology optimization.

[28] Lin C. Y., Kikuchi N., Hollister S. J. (2004). A novel method for biomaterial scaffold internal architecture design to match bone elastic properties with desired porosity. *Journal of Biomechanics*.

[29] Dewi A. H., Ana I. D., Wolke J., Jansen J. (2013). Behavior of plaster of Paris-calcium carbonate composite as bone substitute. A study in rats. *Journal of Biomedical Materials Research—Part A*.

[30] Rojbani H., Nyan M., Ohya K., Kasugai S. (2011). Evaluation of the osteoconductivity of *α*-tricalcium phosphate, *β*-tricalcium phosphate, and hydroxyapatite combined with or without simvastatin in rat calvarial defect. *Journal of Biomedical Materials Research—Part A*.

[31] Roshan-Ghias A., Lambers F. M., Gholam-Rezaee M., Müller R., Pioletti D. P. (2011). In vivo loading increases mechanical properties of scaffold by affecting bone formation and bone resorption rates. *Bone*.

[32] Wagoner Johnson A. J., Herschler B. A. (2011). A review of the mechanical behavior of CaP and CaP/polymer composites for applications in bone replacement and repair. *Acta Biomaterialia*.

[33] Turner T. M., Urban R. M., Gitelis S., Kuo K. N., Andersson G. B. J. (2001). Radiographic and histologic assessment of calcium sulfate in experimental animal models and clinical use as a resorbable bone-graft substitute, a bone-graft expander, and a method for local antibiotic delivery. One institution's experience. *Journal of Bone and Joint Surgery A*.

[34] Glazer P. A., Spencer U. M., Alkalay R. N., Schwardt J. (2001). In vivo evaluation of calcium sulfate as a bone graft substitute for lumbar spinal fusion. *The Spine Journal*.

[35] Murashima Y., Yoshikawa G., Wadachi R., Sawada N., Suda H. (2002). Calcium sulphate as a bone substitute for various osseous defects in conjunction with apicectomy. *International Endodontic Journal*.

[36] Rao P. J., Pelletier M. H., Walsh W. R., Mobbs R. J. (2014). Spine interbody implants: material selection and modification, functionalization and bioactivation of surfaces to improve osseointegration. *Orthopaedic Surgery*.

